# Project MinE: study design and pilot analyses of a large-scale whole-genome sequencing study in amyotrophic lateral sclerosis

**DOI:** 10.1038/s41431-018-0177-4

**Published:** 2018-06-28

**Authors:** Project MinE ALS Sequencing Consortium, Wouter Van Rheenen, Sara L. Pulit, Annelot M. Dekker, Ahmad Al Khleifat, William J. Brands, Alfredo Iacoangeli, Kevin P. Kenna, Ersen Kavak, Maarten Kooyman, Russell L. McLaughlin, Bas Middelkoop, Matthieu Moisse, Raymond D. Schellevis, Aleksey Shatunov, William Sproviero, Gijs H. P. Tazelaar, Rick A. A. Van der Spek, Perry T. C. Van Doormaal, Kristel R. Van Eijk, Joke Van Vugt, A. Nazli Basak, Ian P. Blair, Jonathan D. Glass, Orla Hardiman, Winston Hide, John E. Landers, Jesus S. Mora, Karen E. Morrison, Stephen Newhouse, Wim Robberecht, Christopher E. Shaw, Pamela J. Shaw, Philip Van Damme, Michael A. Van Es, Naomi R. Wray, Ammar Al-Chalabi, Leonard H. Van den Berg, Jan H. Veldink

**Affiliations:** 1 Department of Neurology, Brain Center Rudolf Magnus, University Medical Center Utrecht, Utrecht, The Netherlands; 2 Department of Basic and Clinical Neuroscience, Maurice Wohl Clinical Neuroscience Institute, King’s College London, London, UK; 3 Department of Biostatistics and Health Informatics, Institute of Psychiatry, Psychology and Neuroscience, King’s College London, London, UK; 4 NIHR Biomedical Research Centre at South London and Maudsley NHS Foundation Trust and King’s College London, London, UK; 5 Department of Neurology, University of Massachusetts Medical School, Worcester, MA USA; 6 Genomize Inc. Bogazici University, Technology Transfer Region, ETAB, Istanbul, Turkey; 7 SURFsara, Amsterdam, The Netherlands; 8 Population Genetics Laboratory, Smurfit Institute of Genetics, Trinity College Dublin, Dublin, Ireland; 9 Department of Neurosciences, KU Leuven - University of Leuven, Experimental Neurology and Leuven Research Institute for Neuroscience and Disease (LIND), B-3000 Leuven, Belgium; 10 VIB, Vesalius Research Center, Laboratory of Neurobiology, Leuven, Belgium; 11 Department of Neurology, University Hospitals Leuven, Leuven, Belgium; 12 Neurodegeneration Research Laboratory, Bogazici University, Istanbul, Turkey; 13 Centre for Motor Neuron Disease Research, Faculty of Medicine and Health Sciences, Macquarie University, Sydney, NSW 2109 Australia; 14 Department Neurology, Emory University School of Medicine, Atlanta, GA USA; 15 Emory ALS Center, Emory University School of Medicine, Atlanta, GA USA; 16 Academic Unit of Neurology, Trinity College Dublin, Trinity Biomedical Sciences Institute, Dublin, Ireland; 17 Department of Neurology, Beaumont Hospital, Dublin, Ireland; 18 Biostatistics Department, Harvard School of Public Health, Boston, MA USA; 19 Sheffield Institute for Translational Neuroscience (SITraN), University of Sheffield, Sheffield, UK; 20 Department of Neurology, Hospital San Rafael, Madrid, Spain; 21 Faculty of Medicine, University of Southampton, Southampton, UK; 22 Farr Institute of Health Informatics Research, UCL Institute of Health Informatics, University College London, London, UK; 23 Department of Neurosciences, Experimental Neurology and Leuven Research Institute for Neuroscience and Disease (LIND), KU Leuven - University of Leuven, B-3000 Leuven, Belgium; 24 Queensland Brain Institute, The University of Queensland, Brisbane, Qld Australia

## Abstract

The most recent genome-wide association study in amyotrophic lateral sclerosis (ALS) demonstrates a disproportionate contribution from low-frequency variants to genetic susceptibility to disease. We have therefore begun Project MinE, an international collaboration that seeks to analyze whole-genome sequence data of at least 15 000 ALS patients and 7500 controls. Here, we report on the design of Project MinE and pilot analyses of successfully sequenced 1169 ALS patients and 608 controls drawn from the Netherlands. As has become characteristic of sequencing studies, we find an abundance of rare genetic variation (minor allele frequency < 0.1%), the vast majority of which is absent in public datasets. Principal component analysis reveals local geographical clustering of these variants within The Netherlands. We use the whole-genome sequence data to explore the implications of poor geographical matching of cases and controls in a sequence-based disease study and to investigate how ancestry-matched, externally sequenced controls can induce false positive associations. Also, we have publicly released genome-wide minor allele counts in cases and controls, as well as results from genic burden tests.

## Introduction

Amyotrophic lateral sclerosis (ALS) is a rapidly progressing, fatal neurodegenerative disease [1]. Twin studies estimate the heritability of ALS to be ~60%, suggesting a strong genetic component contributing to disease risk [2], and approximately 10–15% of patients have a clear family history of disease [3]. Genetic risk factors have been extensively studied in familial ALS cases, and this effort has led to the identification of highly penetrant causal variants, including variants residing in *SOD1* [4], *TARBP* [5], *FUS* [6], and *C9orf72* [7, 8]. In so-called sporadic ALS cases, who have no known family history of disease and comprise the majority of all cases, only a small number of other common genetic risk loci have been identified. Among these loci are the *ATXN2* CAG repeat expansion [9], variants in *C21orf2* [10], common variation within the *UNC13A* [11], *SARM1* [12], *MOBP* [10], and *SCFD1* [10] loci and, most importantly, the highly pathogenic *C9orf72* repeat expansion. The latter highlights the typical categorization of “familial” and “sporadic” ALS as likely non-distinct groups with overlapping genetic architectures.

Despite these recent advances in ALS genetics, the bulk of risk loci in ALS remain undiscovered. The most recent and largest ALS GWAS, performed in 12,577 cases and 23,475 controls, showed a disproportionate contribution of low-frequency variants to the overall risk of ALS [10]. Genome-wide association studies to date have focused almost exclusively on common variation (minor allele frequency (MAF) > 1%)), leaving lower-frequency and rarer variants segregating in the general population essentially untested. Thus, there is a pressing need to study rare variation across the full length of the genome in cases with and without family history of disease. To this end, we have begun Project MinE, a large-scale whole-genome sequencing study in ALS. The project leverages international collaboration and recent developments in sequencing technologies, allowing us to explore the full spectrum of genetic variation in samples collected worldwide. Project MinE seeks to obtain sequencing data in 15,000 ALS patients and 7,500 matched controls with the aim of identifying new loci associated to ALS risk, fine-map known and novel loci, and provide a publicly-available summary-level dataset that will enable further genetic research of this and other diseases. A data access committee controls access to raw data, ensuring a FAIR data setup (http://www.datafairport.org).

While common variant association studies have become mostly standardized over the last decade, rare variant association studies such as Project MinE face an array of new challenges. Sequencing studies demand large sample sizes to detect small effects at rare variants, thus making large-scale collaborative consortia a necessity. Sequence data itself, measuring into the terabytes, poses a substantive data storage, processing, and management challenge. The analytic effects of (tight) spatial clustering of rare variants is not yet well understood [13, 14], making careful case/control selection and proper handling of population stratification key. Rare variant association studies typically employ genic burden testing to overcome power problems, thus requiring a series of analytic choices be made regarding the functional annotation [15] and statistical analysis of the data. Here, we discuss the “pilot phase” of Project MinE, performed in 1,264 cases and 611 controls collected in the Netherlands. Though the sample size is too small to detect moderate-effect genetic associations, it allows us to explore the implications of these many analytic challenges faced by Project MinE or any disease study employing sequence data, to understand the genetic basis of disease. We outline the formation of the consortium, study design challenges, data Quality control (QC) and analysis approaches employed by the project, and publicly release all minor allele counts in cases and controls together with results from genic burden tests derived from this dataset.

## Materials and methods

### Consortium design

**Fig. 1:**
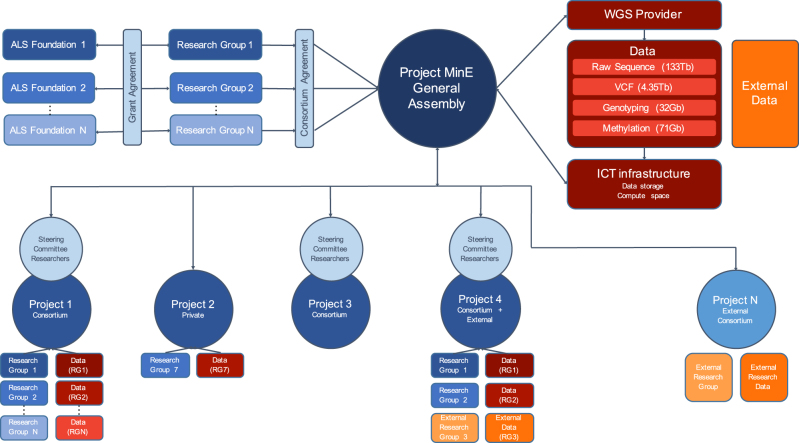
Consortium design. Consortium structure including funding agencies, research groups, the Project MinE general assembly, sub-projects, and data management. Each research group obtains funding using uniform grant proposals shared between the research groups. DNA samples are provided to the sequencing providers via the Project MinE general assembly. Samples are sequenced, and data is centrally stored (the size of the data for the first 1,935 samples is indicated in parentheses; the full dataset for *N* = 22,500 samples will be ~1.7 petabytes). External data can also be contributed to and integrated into the dataset. Different research groups, including external groups, collaborate to work on specific projects. ALS amyotrophic lateral sclerosis, WGS whole-genome sequencing

The Project MinE consortium includes ALS research groups from 16 different countries collaborating in a “franchise” design (www.projectmine.com, Fig. 1). This design means that samples and sample-affiliated data (e.g., dense phenotyping) from partners are collected, processed and stored according to the same protocol, while partners maintain full control over their samples, affiliated data, and any additional data generated on those samples. Within the consortium, ALS patients and controls are ascertained through clinics affiliated to the research groups. At these clinics, neurologists obtain a standardized core clinical data set with phenotypic information (Supplementary Table 1) and blood is drawn for DNA isolation. DNA samples are stored on site, but are collectively prepared for sequencing at commercial sequencing providers, in batches of up to 1,000 samples, so research groups can profit from a lower pricing scheme available when large numbers of DNA samples are provided together.

A number of variables, including pricing for varying numbers of genomes, DNA input requirements, availability of PCR-free library preparation, and methods for data delivery, were all considered during selection of a sequencing provider (Supplementary Table 2). The majority of the data to date have been sequenced at Illumina (San Diego, CA, USA); a small subset is currently being sequenced locally in Australia and Canada. From Illumina, the data are transferred through the internet (via a secure connection) to SURFsara (Amsterdam, The Netherlands). After arrival, the data is automatically checked for corruption and stored in duplicate on two geographically separated tape silos. The storage system is connected to a distributed parallel file system (dCache [16]) that provides high performing file service to a 5,600 core high-throughput computing cluster.

From here, consortium members can access the genomes of their samples and ask that their data be additionally backed up on the High-Performance Compute cluster at the University Medical Center Utrecht (Utrecht, The Netherlands). Analysis teams (possibly including researchers from external groups) can submit analysis proposals to the consortium and then gain access to all genomes and make use of the SURFsara compute facilities (Fig. 1). Access to the data or usage of the compute facilities is contingent on (a) X.509 authentication certificates and (b) proposed analyses for the data. This data infrastructure allows, for example, particular cohorts and individuals access to particular subsets or all of the data, depending on the analysis. Based on permission given by the manager of the data (i.e., the principal investigator of the contributing research group), access is provided through the gridftp protocol v2 that is tailored for large and reliable file transfers. The X.509 certificates allow for the possibility to scale out to other compute facilities; given valid credentials, the data can then be accessed from anywhere.

### Sample selection

Patients were diagnosed with definite, probable, and probable lab-supported ALS according to the revised El Escorial Criteria [17]. For the pilot analyses presented here, all patients were seen by neurologists specialized in motor neuron diseases at the University Medical Center Utrecht and Academic Medical Center (Amsterdam, The Netherlands). The samples were all included in the Prospective ALS study in the Netherlands (PAN), an incidence-based registry of ALS cases [18]. Controls were population-based controls that were matched for age, sex, and geographical region. As is true for all contributing cohorts to Project MinE, cases and controls were ascertained in a roughly 2:1 ratio; the 2:1 case-control ratio was selected to improve detection of variants in cases and with the plan to include publicly-available sequenced controls in future analyses.

### Phenotypic information

For all participants in Project MinE, we have defined a core clinical dataset, meant to be collected and made available for all cases (Supplementary Table 1). Phenotypic information is stored physically separately (https://euromotor.umcutrecht.nl) from the genetic data in a clinical database called Progeny. The phenotypic and genetic data can be connected through sample identifiers. Phenotypic data storage is organized similarly to the sequencing data: every contributing group has default full access to their own phenotypic data, but data can easily be jointly shared analysed if desired. This core clinical dataset includes date of birth, sex, site of onset, date of disease onset, diagnostic category, Forced Vital Capacity at time of diagnosis, cognitive status, and a revised ALS functional rating scale score [19]. The required endpoint data include date of death, date of starting invasive ventilation or requiring continuous non-invasive ventilation. For the pilot data used in this study, we obtained additional information on place of birth through the Dutch Municipal Personal Records Database.

### Whole-genome sequencing

Venous blood was drawn from patients and controls from which genomic DNA was isolated using standard methods. We set the DNA concentrations at 100 ng/μl as measured by a fluorometer with the PicoGreen® dsDNA quantitation assay. DNA integrity was assessed using gel electrophoresis. All samples were sequenced using Illumina’s FastTrack services (San Diego, CA, USA) on the Illumina HiSeq 2000 platform. Sequencing was 100 bp paired-end performed using PCR-free library preparation, and yielded ~40× coverage across each sample. The Isaac pipeline [20] was used for alignment to the hg19 reference genome as well as to call single nucleotide variants (SNVs), insertions and deletions (indels), and larger structural variants (SVs). Both the aligned and unaligned reads were delivered in binary sequence alignment/map format (BAM) together with variant call format (gVCF) files containing the SNVs, indels and SVs [20]. gVCF files were generated per individual and variants that failed the Isaac-based quality filter were set to missing on an individual basis.

### Data analysis

#### Quality control

QC of the data included QC at an individuals and variant level. Full details of QC are provided in the supplement.

#### Principal component analysis

Principal components were calculated for all individuals including variants at different allele frequency thresholds using GCTA [21]. The eigenvectors of the first twenty principal components were regressed on latitude and longitude of birthplace. In a leave-one-out scheme, this linear model was used to predict the birthplace for the individual left out.

#### Identity-by-descent analysis

We phased all non-singleton variants using SHAPEIT2 [22] and then used BEAGLE4 [23] to detect runs of identity-by-descent (IBD) between individuals.

#### Association analysis

Genotypes at common variants (MAF > 0.5%) were tested for an association with case/control status using logistic regression (PLINK v1.9) [24, 25] assuming an additive model. Optionally, the first ten principal components (PCs) were included as covariates. To test rare variation, we performed genic “burden” testing. All variants were functionally annotated using ANNOVAR [26]. We then determined three functional groups for gene-based association testing: (a) loss of function (LOF) variants (premature stop variants, stop-loss variants, variants at splice sites, and frameshift indels), (b) nonsynonymous variants, and (c) LOF and nonsynonymous variants (aggregated). Burden testing (T1, T5, Variable Threshold, Madsen-Browning and SKAT) was implemented using ScoreSeq [27] and performed across all variants with MAF < 5%. All burden tests were adjusted for sex and the top ten PCs and performed on the QC-passing set of unrelated samples (1169 cases and 608 controls).

#### Population stratification and externally sequenced controls

To assess the type I error in a burden testing framework, we simulated 100 different phenotypes for two scenarios: perfect matching and imperfect matching with a North-to-South gradient for the number of cases (keeping the case/control counts the same, Supplementary Figure 1), and then performed burden testing on LOF and nonsynonymous variants with and without principal components as covariates. The most extreme *p*-value from each simulation was extracted (resulting in 100 extreme *p*-values total, per scenario); the fifth-most extreme *p*-value represented the *p*-value threshold necessary to maintain study-wise type I error at 5%. Subsequently, we assessed the impact of including ancestry-matched, externally sequenced controls (the Genome of the Netherlands, GoNL [14]) instead of controls sequenced as a part of Project MinE. As their data is mostly available formatted as VCF files, we combined both datasets on a VCF level, without realignment or joint variant calling. The structure of this dataset was described by principal component analysis and we assessed inflation of the test statistics when externally sequenced controls were included in genome-wide burden testing.

### Study approval and informed consent

All participants gave written informed consent and the institutional review board of the University Medical Center Utrecht approved this study. Additional approval was obtained to access the Dutch Municipal Personal Records Database.

### Data access

The summary statistics generated in this study are available at the Project MinE databrowser: http://databrowser.projectmine.com/.

## Results

### Baseline characteristics and description of data

**Fig. 2:**
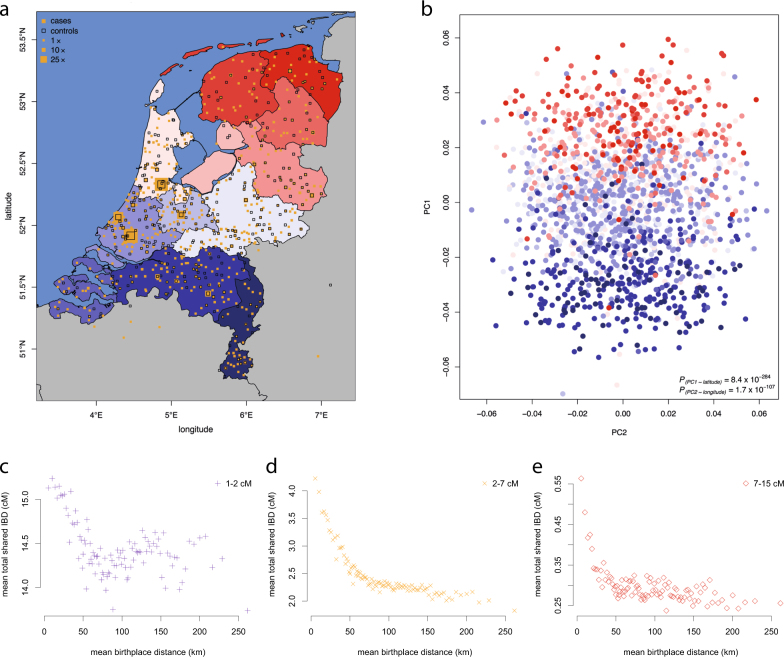
Population structure by principal component and IBD analysis. **a** Birthplaces of cases and controls for individuals born in The Netherlands. **b** The first two principal components reflect the geographical distribution of samples. **c–e** Relation between birthplace distance between pairs of individuals and shared IBD segments 1–2 cM (**c**), 2–7 cM (**d**) and 7–15 cM (**e**). PC principal component, *MAF* minor allele frequency, *IBD* identity by descent, *km* kilometer, *cM* centimorgan

**Fig. 3:**
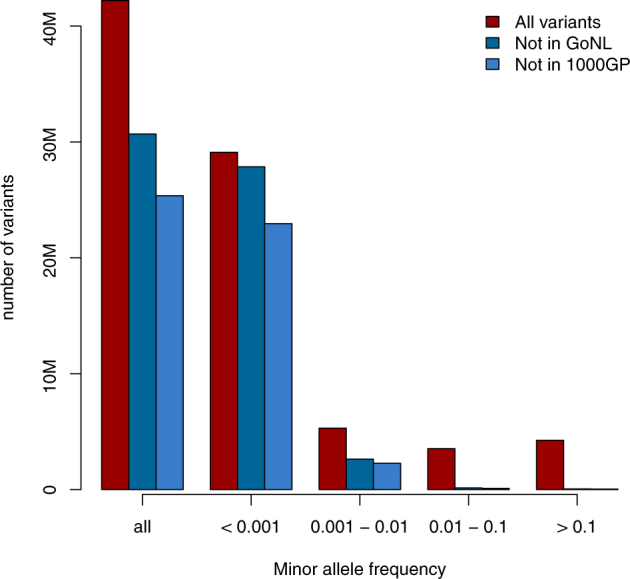
Allele frequency distribution and comparison to existing datasets. The majority of rare variants (<1%) were not found in public datasets such as the Genome of the Netherlands and the 1000 Genomes Project phase 1. In contrast, almost all common alleles (>10%) were found in these datasets. GoNL Genome of the Netherlands, 1000GP 1000 Genomes Project

After QC, 1,169 unrelated Dutch-ancestry cases and 608 ancestrally-matched controls were available for analysis. Their baseline characteristics are displayed in Table 1 and the distribution of cases and controls throughout The Netherlands is displayed in Fig. 2a. In total, 42,200,214 SNVs and indels passed QC. The majority (69%) of these sequenced variants were rare (MAF < 0.001, <3 allele observations in our dataset, Fig. 3). In particular, the bulk of these rare variants were not observed in publicly available datasets of whole-genome sequencing data including the Genome of The Netherlands project (GoNL release 5 from 28-10-2013, *N* = 498) and the 1000 Genomes Project (Phase 1 from 23-11-2010, *N* = 1,093). This observation reflects population-specific variants and the growing number of rare variants that will continue to be discovered as sequencing is performed in increasingly larger samples around the globe. As expected, most common variants (MAF > 1%) have been observed in these two datasets (97.5% in GoNL and 98% in the 1000 Genomes Project) reflecting global sharing of common variation.

**Table 1:** Baseline characteristics. Baseline characteristics of samples included in Project MinE

	ALS	Controls
*N*	1,196	608
Sex, % female	41.1	43.1
Type		
Familial (%)	31 (2.6)	–
Sporadic (%)	1,165 (97.4)	–
C9orf72		
WT (%)	1,095 (91.6)	–
Expanded (%)	74 (6.2)	–
Unknown (%)	27 (2.3)	–
Age at onset, years (IQR)	64.0 (56.6 - 70.1)	–
Site of onset		
Bulbar (%)	386 (32.3)	–
Spinal (%)	718 (60.0)	–
Thoracic (%)	30 (2.5)	–
Unknown (%)	62 (5.2)	–
Survival, years (IQR)	2.29 (1.58–3.28)	–
Deceased (%)	966 (80.8)	–

*WT* wild type, *IQR* interquartile range

### Geographic clustering

The first and second principal components reflected the geographical distribution of cases and controls in detail (Fig. 2b). The eigenvectors of the first principal component explained 55% of the variance in latitude (*p* = 8.4 × 10^−284^) and the second principal component explained 24% of the variance in longitude (*p* = 1.7 × 10^−107^) of the geographical distribution of the samples across the Netherlands. Prediction models including the first twenty principal components predicted the birthplace of individuals with high accuracy (Supplementary Figure 2). The accuracy increased when including rarer variants; the median distance between the predicted and actual birthplace decreased from 50 km (31 miles) when considering common variants (MAF > 0.1), to 36 km (22 miles) when including rarer variants (MAF > 0.001), illustrating the strong geographical clustering of rare variants [28].

Sharing of IBD segments showed strong geographical patterns throughout the Netherlands. As was observed in a previous population genetics study in The Netherlands there is a clear North-to-South gradient for sharing of shorter (and thus older) IBD segments (Supplementary Figure 3a). We also confirmed the finding that people born in the Southern provinces shared more of these IBD segments with people from the Northern provinces than they share with people within their own province, reflecting the previously described complex migration patterns [14]. Sharing of longer IBD segments, reflecting more recently shared ancestry, was highly dependent on the distance of birthplaces between individuals (Fig. 1c, e and Supplementary Figure 3b).

### Association testing in ALS cases and controls

We found no variants reaching genome-wide significance (*p* = 5.0 × 10^−8^) with ALS in our sample. We looked up known associated single-variants, three of which showed nominal association to the trait (*p* < 0.05, Supplementary Table 3). Similarly, no loci achieved exome-wide significance in burden testing (*p* ≈ 5.0 × 10^−7^, after adjusting for 20,000 tested genes, the various burden test types, and sets of SNPs tested; Supplementary Figure 4) due to limited power (Supplementary Figure 5). Burden testing *p*-values for all genes tested can be found at http://databrowser.projectmine.com/.

### The implications of study design

We sought to evaluate how matching cases and control, thereby introducing fine-grained population structure, influenced the burden testing type I error rate. Therefore, we simulated phenotypes that (1) created perfectly geographically matched case-control sets and (2) introduced population structure between patients and controls (Supplementary Figure 1). We subsequently ran simulations that did not correct for any covariates or those that included common-variant (MAF > 0.1%) principal components. Imperfect matching did not yield a markedly more stringent p-value to maintain a study-wide type I error at 0.05 for all burden tests (Table 2), likely due to limited power. Correcting for PCs did not affect the *p*-value to maintain study-wise type I error at 0.05 either.

**Table 2:** Population structure and type I error. No marked differences were observed between the *p*-values in perfect or imperfect matching scenarios (with or without PCs) to maintain the study-wise type I error rate at 0.05

Scenario 1: perfect matching				
	Type I error in burden tests			
	T1	MB	VT	SKAT
No PCs	4.25 × 10^−6^	3.38 × 10^−6^	3.94 × 10^−6^	2.35 × 10^−6^
Common PCs	2.86 × 10^−6^	3.99 × 10^−6^	8.12 × 10^−6^	2.24 × 10^−6^

Scenario 2: imperfect matching				
	Type I error in burden tests			
	T1	MB	VT	SKAT
No PCs	4.40 × 10^−6^	1.90 × 10^−5^	1.46 × 10^−5^	6.72 × 10^−6^
Common PCs	5.35 × 10^−6^	9.35 × 10^−6^	1.04 × 10^−9^	7.81 × 10^−6^

To test the analytic implications of a study design that uses its resources solely for the sequencing of cases and collects controls from an external source, we merged genotypes from the ALS cases with Dutch-ancestry samples whole-genome sequenced as part of the Genome of the Netherlands (GoNL) project [14]. Principal component analysis indicated a clear separation of the two projects, where PC1 effectively captured each project (Supplementary Figure 6a). Removal of highly differentiated SNPs with a frequency difference > 5% across the two projects somewhat mitigated this separation (Supplementary Figure 6b). As expected, single-variant testing between ALS cases and GoNL controls revealed excessive genomic inflation (*λ* = 1.12). Similarly, burden testing comparing ALS cases and GoNL controls revealed a strongly inflated QQ plot and genomic inflation factor (*λ* = 2.49), demonstrating the challenges of using externally sequenced controls to identify disease genes in a separately-sequenced set of cases and controls.

## Discussion and future perspectives

Family-based and population-level genetic studies have revealed ALS as a complex disease with a distinct role for rarer genetic variation. Although numerous genetic variants have been identified as conferring ALS risk, the genetic basis in the vast majority of cases is not yet understood. Here, we have described the design and pilot analyses of a large-scale whole-genome sequencing study aimed at discovering new genetic risk factors and further elucidating the genetic basis of ALS.

Data sharing and transparency in scientific research is advantageous for a host of reasons: it allows for analyses across large sets of samples, particularly for lower-prevalence diseases such as ALS; ensures rigorous experiments that can be reproduced by external groups; and allows for publicly-funded research to be made available to the public itself. Project MinE cannot publicly release individual-level genotype data due to consent. However, genotype frequency information and genic burden testing results are publicly available at the project’s online browser (http://databrowser.projectmine.com/). This browser will be continuously updated as the project expands, and will provide more detailed data integration in future releases. To further enhance the transparency within the consortium itself and with external collaborators and researchers, the project has begun a GitHub repository, to share and maintain scripts and data processing pipelines.

Given the relatively low prevalence of ALS, international collaboration is crucial to assembling large samples. Thus far, the “franchise” design of our consortium has allowed us to rapidly expand to sixteen participating research groups globally. This joint effort has collected enough resources to sequence > 7000 samples, with more currently being collected. Ongoing analyses include case-control association testing, as well as association testing in age-of-onset, and survival time. Additionally, the available core clinical dataset, uniformly collected for all individuals included in Project MinE, will allow for a host of other discovery analyses.

The pilot analyses, though small in sample size, yield several immediate and important conclusions. First, while genotype-called data (in VCF format) is easily handled by a high-performance compute cluster, whole-genome BAM files (approximately 80 gigabytes per genome) demand a compute infrastructure that can handle terabytes and even petabytes of data. This includes direct delivery of the genomes that are currently being sequenced via a direct high-speed connection between the sequencing provider and a computer cluster with a GRID architecture (SURFsara). Though some research institutions are already prepared for such a deluge of data, others will need to carefully consider compute infrastructure and the technical ramifications of assembling big data before embarking on large-scale sequencing analyses. Further, having all data stored in a single location enables analysis across the full dataset, including on BAM-level data.

Second, initial QC of the data suggests that the high-depth sequencing and downstream genotype calling is yielding high-quality data, reflected in our ability to reconstruct, through principal component analysis and identity-by-descent analyses, demographic observations that have been made in separate large-scale sequencing efforts in the Dutch population [14]. Further, the population analyses performed here and in particular the birthplace analysis, highlights the sensitivity of genome-wide sequence data. The data must be carefully protected such that those who have donated DNA to enable disease research, will also remain anonymous (in accordance with the consent provided) [29].

Third, the high-resolution geographical clustering of rare variants as observed in these analyses could pose challenges in finding rare variants that contribute to ALS risk. The commonly applied burden test approach, however, does not seem to suffer from increased type I error rates induced by modest imperfections in geographically matching samples, as was shown by the burden test simulations. While it is possible that aggregating rare variants (found in potentially many geographic locations) in these tests makes the approach more robust to population structure, these results should be interpreted with caution. The modest power in our pilot sample likely limited our ability to detect the increasing type I error rates induced by imperfect geographic sample matching. With the increasing number of ancestrally diverse samples that are being included in Project MinE, false positive associations due to population stratification remain a serious concern, especially when our aim becomes identifying the causal single variant(s) that drive the genic burden signal. Correcting for principal components can help to control type I error, though potentially not sufficiently so in burden testing. More sophisticated analytic approaches, such as employing a linear mixed model in single variant and genic burden testing, will likely be helpful in controlling for population stratification across the full set of Project MinE samples [30].

We further demonstrate that inclusion of externally-sequenced samples can pose analytical challenges. Specifically, we show that using ancestry-matched controls from publicly-available datasets can strongly inflate association test statistics if the data is simply combined at VCF-level. Sequence data can contain underlying structure due to sequencing platforms, coverage, alignment and calling algorithms. Inclusion of externally sequenced cases and controls (sequenced either separately or together) is non-trivial must be approached with extreme rigor. Methods for handling the merging and calling of heterogeneous sequencing datasets at a BAM-level are currently being applied in large consortia [31–33] and indicate that obtaining a high-quality dataset requires calling genotypes from petabytes of raw data in a uniform way across the full set of samples. Additional developments in handling heterogeneous sequence data include association testing on read-level data to allow for externally sequenced controls [34]. Although these methods come with significant computational burden, similar approaches could be applied in Project MinE, in an effort to expand beyond the 22,500 samples sequenced initially by the project.

With the veritable explosion in methods and approaches being developed for whole-genome sequence data analysis, Project MinE will seek to leverage new and powerful methods for uncovering ALS risk variants. Current plans include more sophisticated functional annotation techniques, such as unsupervised-learning approaches to discriminate between functionally relevant and benign variation in the genome [35, 36]; implementing a linear mixed model approach to burden testing [30]; a burden testing framework that will focus not only on genes but also on regulatory and other non-coding elements; and analyses that investigate both variation and methylation data. We have additionally established a pipeline to lift all our data to the newest genome build (hg38), which will likely yield better coverage of the genome in our samples. As the size of the data expands and additional collaborators join the effort, we will explore additional data structures that can facilitate transcontinental data analysis with minimal physical movement of data.

Finally, in addition to testing SNVs and indels, we will explore the role of more complex genetic variation such as structural variants and repeat expansions, which are known to play an important role in ALS susceptibility, yet have never been studied at genome-wide scale. With a global collaboration in place, a wealth of genetic variation being generated, and new methods for sequence data constantly in development, Project MinE will be the largest and most complete study of ALS genetics to date, poised to reveal novel risk loci, fine-map known disease genes, and shed light on the biological drivers of disease.

## Electronic supplementary material

Supplementary material
