# Supplementary material for: Project MinE: study design and pilot analyses of a large-scale whole-genome sequencing study in amyotrophic lateral sclerosis
Source: Eur J Hum Genet. 2018 Jun 28;26(10):1537–46. doi: 10.1038/s41431-018-0177-4 (PMC6138692; doi:10.1038/s41431-018-0177-4)
Supplement: Supplementary file 1 — Supplementary material [file 41431_2018_177_MOESM1_ESM.docx]

# **Supplementary Information**

# **Project MinE: study design and pilot analyses of a large-scale whole genome sequencing study in amyotrophic lateral sclerosis**

## Supplementary Figures


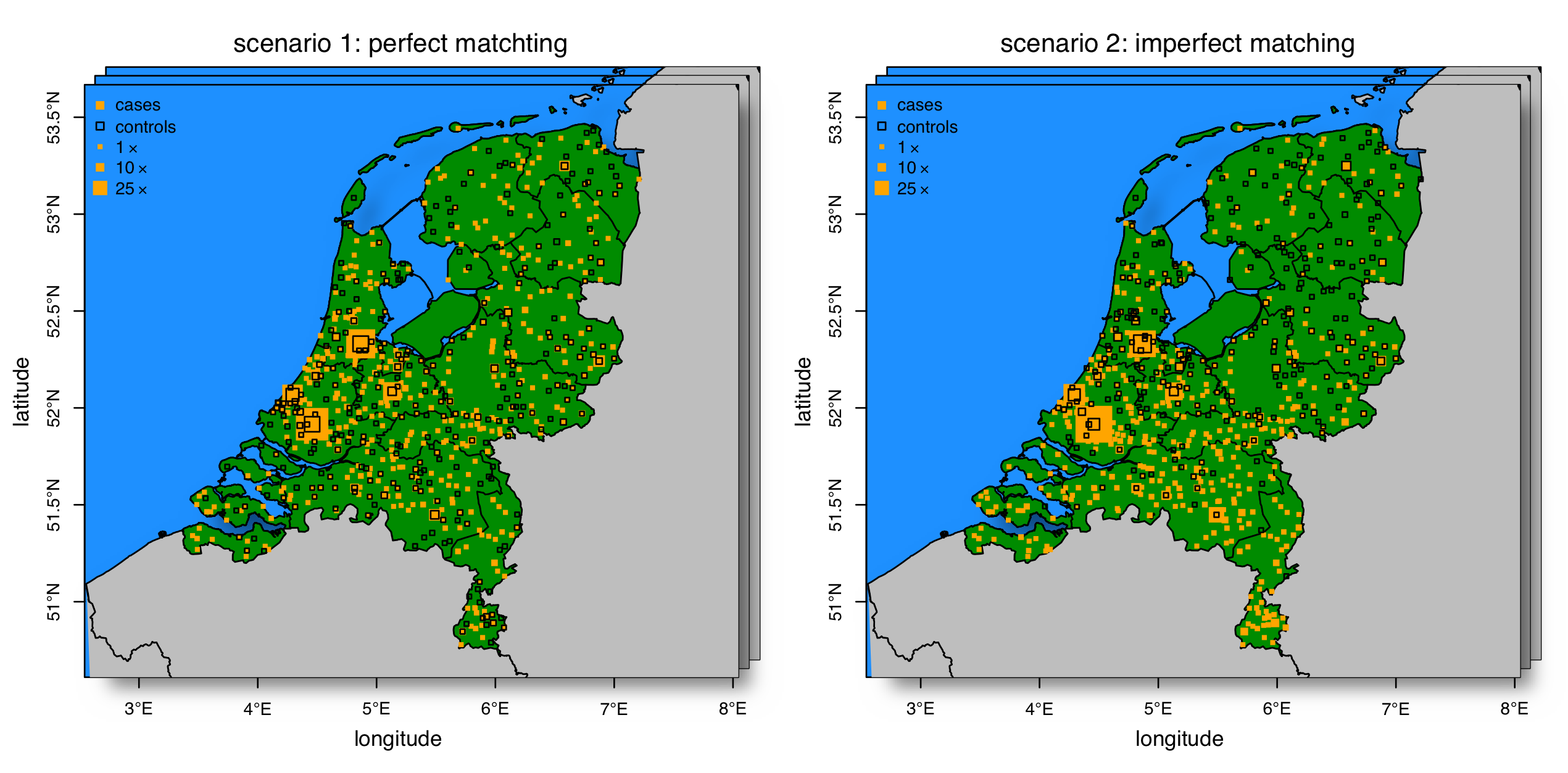


**Supplementary Figure 1. Simulated phenotypes**. Simulations were performed under the null-hypothesis of no genetic association in two different scenarios: perfect matching of cases and controls, which is equal to a random permutation of the phenotype (left panel) and imperfect matching in which we varied the number of cases and controls drawn from specific geographic regions following a North-to-South gradient (right panel). For both scenarios 100 phenotypes were simulated.


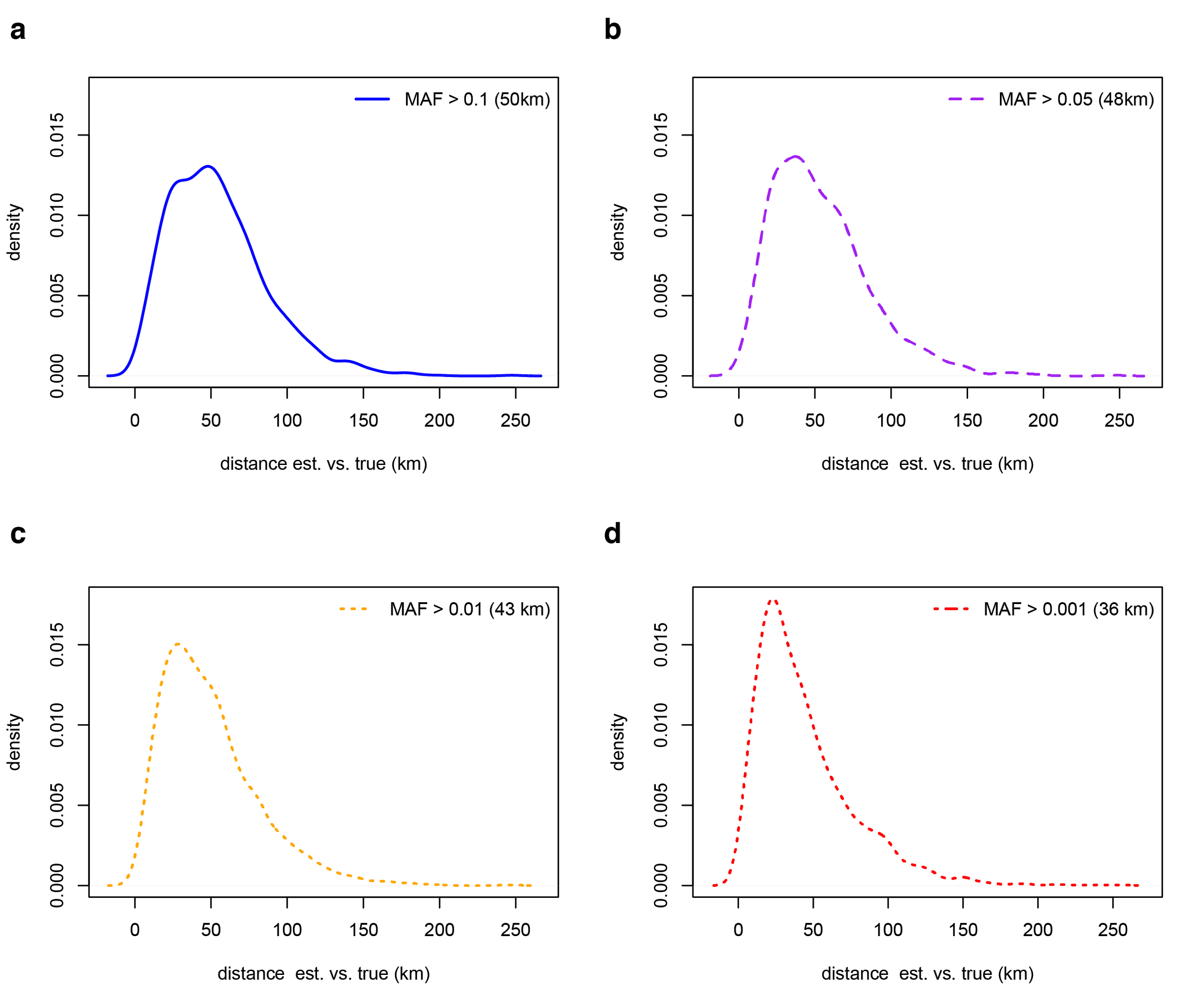


**Supplementary Figure 2** Distribution of distance between actual and predicted birthplace based on principal components in a leave-one-out scheme. Predictions were more accurate when more low frequency variants were included in the principal component analysis: median distance between actual and predicted birthplace was 36 kilometres when variants with a minor allele frequency > 0.001 were included. PC, principal component, MAF, minor allele frequency, km, kilometre.


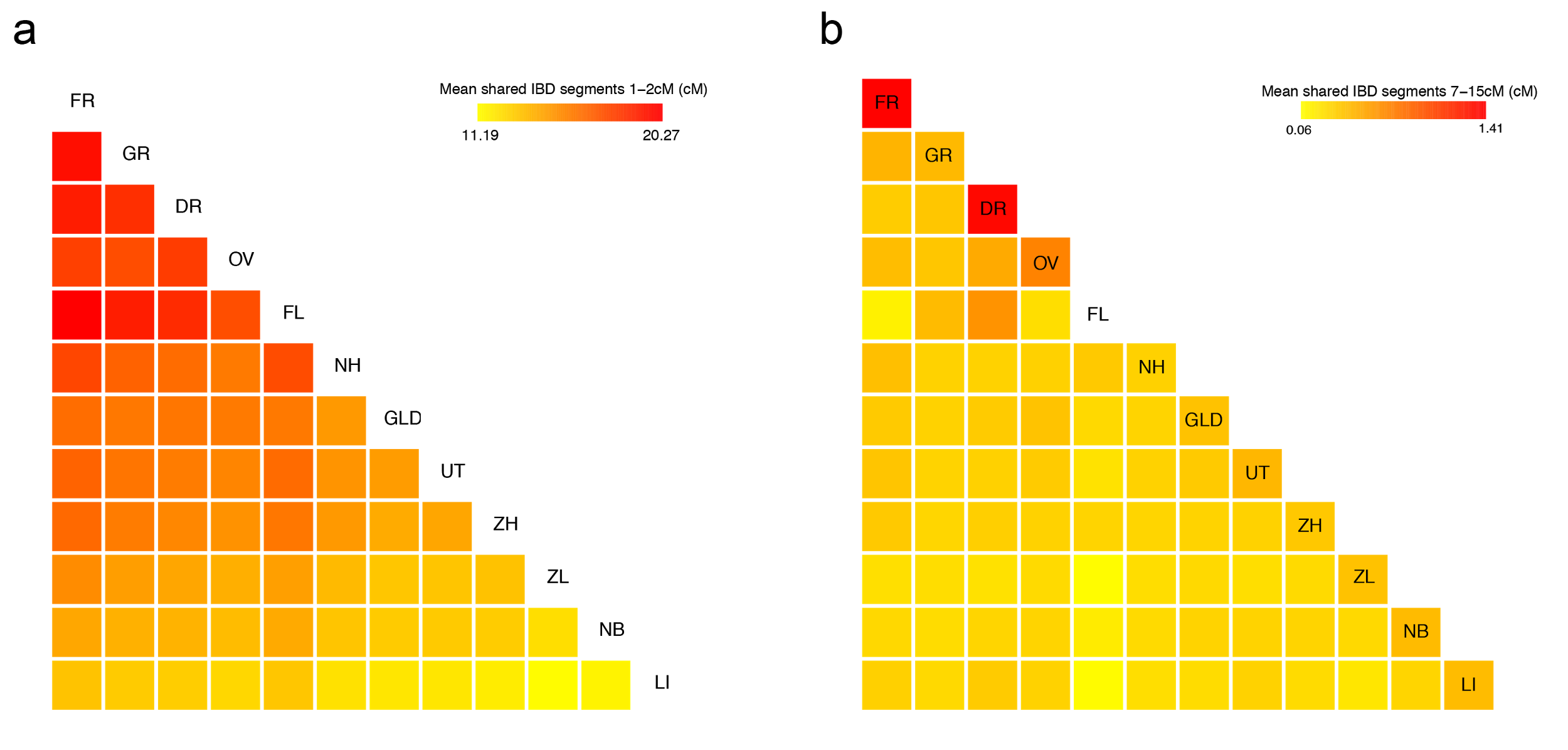


**Supplementary Figure 3** IBD sharing between individuals from different provinces (ordered along the diagonal from North to South). The shorter, older, IBD segments (1 - 2 cM) are mostly shared between the Northern provinces, and interestingly all individuals regardless of the province of birth share more IBD with Northern provinces than the Southern provinces. The longer IBD segments, indicating more recent shared ancestry, are predominantly shared between individuals from the same province. FR, Friesland; GR, Groningen; DR, Drenthe; OV, Overijssel; FL, Flevoland; NH, Noord Holland; GLD, Gelderlands; UT, Utrecht; ZH, Zuid Holland; ZL, Zeeland; NB, Noord Brabant; LI, Limburg; cM, centimorgan.


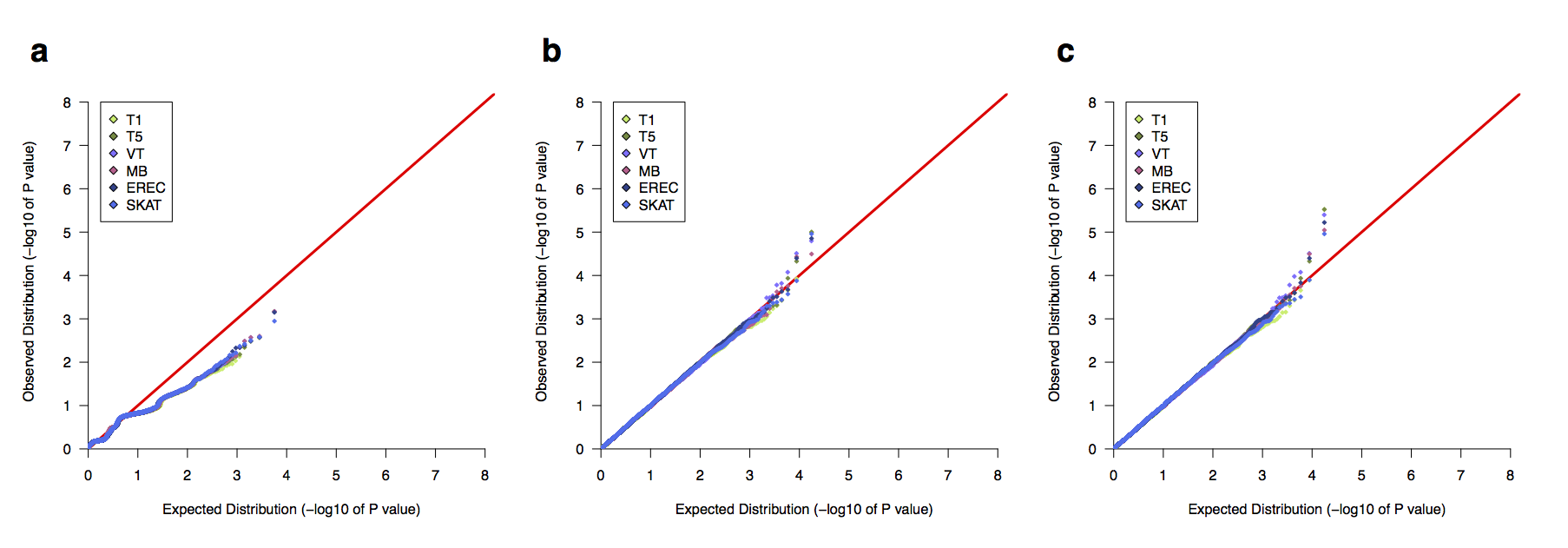


**Supplementary Figure 4** Quantile-quantile (QQ) plots for all burden tests performed in the pilot data. Burden testing was performed in three functional groups: (**a**) loss of function only, (**b**) nonsynonymous only, and (**c**) loss of function and nonsynonymous variation combined. The following tests were performed: T1, test of variants with frequency <1%; T5, test of variants with frequency <5%; MB (Madsen-Browning) test inversely weights variants by frequency; VT (Variable Threshold), tests various frequency cut-offs and then performs multiple test correction; SKAT (Sequence Kernel Association Test), association test that allows for risk and protective variants. Genome-wide results of burden tests (i.e., for all genes) can be found at http://databrowser.projectmine.com/.

**Supplementary Figure 5** Power analyses for the SKAT burden test. We ran simulations using the SKAT library (implemented in R), which contains 10 000 haplotypes for power simulations. Simulations were run using the Power_Logistic command. Power was simulated for a binary phenotype assuming that the percentage of causal variants (Causal.Percent argument in R) in the gene ranged from 10 - 100% and that the region (i.e., gene) was 5 000 basepairs long (SubRegion.Length=5000). The percent of causal variants is noted in the header of each figure. For each setting of causal variants, we additionally simulated a percentage of variants that do not associate with disease (i.e., are null). Power to discover the gene at p = 1 x 10^-6^ is shown in each of the plots for each of the parameters described. Sample sizes (total N) assume a 1-to-1 case-control ratio. For each set of parameters, we ran a total 100 simulations (N.Sim=100), assuming that the maximum odds ratio of a variant in the gene was 3 (MaxOR=3; **a**) or 7 (MaxOR=7; **b**).


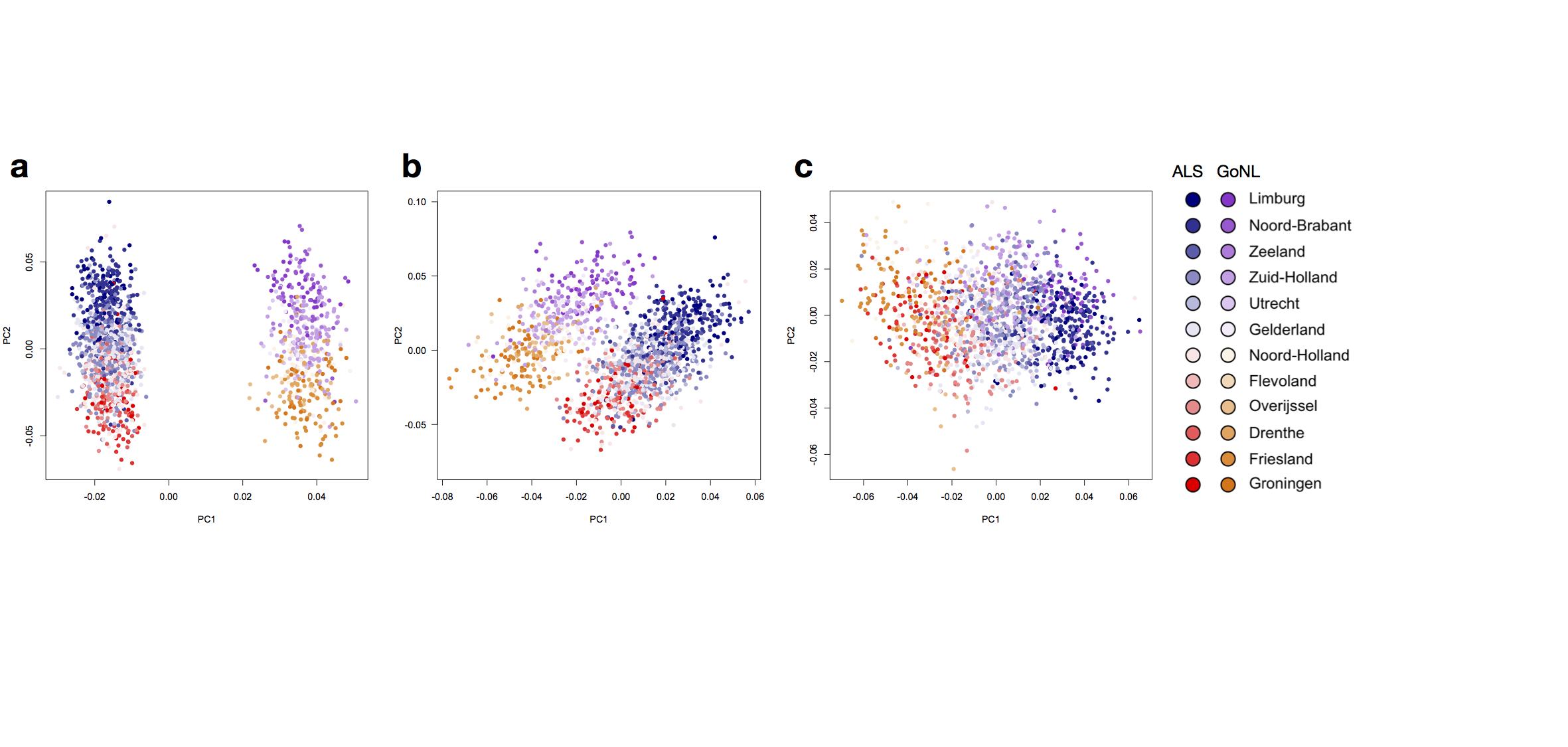
**Supplementary Figure 6** Principal component analysis (PCA, using EIGENSTRAT) in the dataset comprised of both the ALS samples as well as unrelated samples sequenced as part of the Genome of the Netherlands (GoNL) Project. All PC analyses used SNPs that were LD pruned at an r^2^ threshold of 0.2, not lying in the MHC region and LCT locus and inversions on chromosomes 8 and 17, not A/T and C/G SNPs and were in the autosome. The legend indicates the color-coding, based on the province in the Netherlands from which the sample was drawn. **(a)** PCA using only common variation (frequency > 5%). **(b)** PCA using only common variation and removing highly-differentiated SNPs with an allele frequency difference > 5% across the two studies. **(c)** PCA using low-frequency variation (frequency 1 – 5%).

| Information/variable | Details, if applicable |
| --- | --- |
| Sex | Male, female, NA |
| ALS or FTD | As defined by El Escorial if available |
| Revised diagnosis? | Report date and alternative diagnosis |
| Date of birth | - |
| Date of death | - |
| Date of check-living status | - |
| Date of disease onset | - |
| Age at inclusion | - |
| Date of diagnosis | - |
| Date of blood draw for DNA | - |
| Site of onset | Applicable if phenotype is ALS. Defined as: arm OR leg OR bulbar OR spinal OR thoracic/respiratory |
| Forced vital capacity (FVC) | In percentage of expected |
| C9orf72 status | Expanded, normal, not tested, failed |
| SOD1 | Normal, mutated, not tested, failed |
| FUS | Normal, mutated, not tested, failed |
| TARDBP | Normal, mutated, not tested, failed |
| ALSFRS-R | Score at diagnosis |
| Date of ALSFRS-R | - |
| Cognitive score | ECAS total score |
| FTD | Defined as: behavioral FTD or executive FTD OR no FTD |
| Family history | 1st and/or 2nd degree ALS, 1st and/or 2nd degree FTD, 1st and/or 2nd degree ALS/FTD |
| Ethnicity | E.g., Caucasian, Asian, Latin American, etc. |
| Parent 1 ID | - |
| Parent 2 ID | - |
| Matched sample ID | For twins |
| Intended project | If initially intended for another project, flag for new consent to be asked at the institute of origin |

## Supplementary Tables

**Supplementary Table 1** Information collected as the ‘core clinical set’ in Project MinE. FTD, frontotemporal dementia; FVC, forced vital capacity; ECAS, Edinburgh Cognitive and Behavioral ALS Screen; ALSFRS-R, revised ALS functional rating scale score.

| Metric/check | Details |
| --- | --- |
| Pricing | Price per genome at a minimum 30x coverage for 4 000 or 5 000 genomes |
| **Sample submission** | |
| DNA input requirements | E.g., amount (ug) of DNA necessary, with and without additional genotyping on a SNP array |
| Experience with working with sequencing consortia | - |
| Accepts samples from multiple contributors | - |
| **Data generation** | |
| Sequencing platform(s) used | - |
| Library preparation kit used | - |
| Genotyping data on each WGS sample | Which arrays, additional costs, additional DNA |
| Use LIMS | - |
| Positive sample tracking with automation | - |
| Dedicated QC management for End-to-End workflow | - |
| **Data analysis** | |
| Algorithms used | For alignment, variant calling, structural variant calling, copy number variant calling, variant annotation |
| Data file format | FASTA, BAM, CRAM, (g)VCF, etc. |
| Project QC file format | - |
| Reference genome utilized | - |
| Provides software versioning information | - |
| Manages software versioning | - |
| **Data delivery** | |
| Method for data delivery | Hard-drives or direct internet connection |
| Sample TAT | For e.g., 5 000 genomes at 30x coverage |
| Average file size per genome | - |
| **Miscellaneous** | |
| Experience | - |
| Invoicing process | - |
| **Additional services** | |
| Phasing analysis add-on | - |
| Methylation array | - |
| Downstream application support | for targeted panels, array fine-mapping |
| Are softwares and algorithms publicly available? | for collaborators not using the sequencing provider |

**Supplementary Table 2** The checklist used to compare various sequencing providers during provider selection.

| **SNP** | **Gene** | **CHR** | **BP** | **A1** | **OR** | **P** |
| --- | --- | --- | --- | --- | --- | --- |
| rs6700125:T>C | *FGGY* | 1 | 59702797 | T | 1.08 | 0.3172 |
| rs6703183:T>C | *CAMK1G* | 1 | 209712889 | C | 1.019 | 0.8032 |
| rs4674345:A>G | *CYP27A1* | 2 | 219671598 | A | 0.9678 | 0.6381 |
| rs616147:G>A | *MOBP* | 3 | 39534481 | A | 1.116 | 0.1638 |
| rs2708909:G>T | *SUN3* | 7 | 48051679 | G | 0.9831 | 0.8132 |
| rs2708851:G>A | *C7orf5* | 7 | 48085802 | G | 0.9666 | 0.6391 |
| rs10260404:T>C | *DPP6* | 7 | 154210798 | C | 1.028 | 0.6972 |
| rs7813314:C>G | -- | 8 | 2415366 | C | 0.9 | 0.3691 |
| rs12546767:T>C | *8q24.1* | 8 | 126082565 | C | 0.895 | 0.3551 |
| **rs3849943:C>T** | ***C9orf72*** | **9** | **27543382** | **C** | **1.194** | **0.02824** |
| rs2306677:A>G | *ITPR2* | 12 | 26636386 | A | 1.046 | 0.7364 |
| rs74654358:G>A | *TBK1* | 12 | 64881967 | NA | NA | NA |
| rs10139154:C>T | *SCFD1* | 14 | 31147498 | T | 0.9754 | 0.742 |
| **rs7477:A>C** | ***CENPV*** | **17** | **16246016** | **A** | **1.183** | **0.01737** |
| rs35714695:G>A | *SARM1* | 17 | 26719788 | A | 0.8419 | 0.06672 |
| rs1788776:G>A | *18q11.2* | 18 | 21244037 | G | 1.028 | 0.6991 |
| **rs12608932:A>C** | ***UNC13A*** | **19** | **17752689** | **C** | **1.32** | **0.0001982** |
| rs75087725:C>A | *C21orf2* | 21 | 45753117 | A | 1.699 | 0.08897 |
| rs8141797:A>G | *SUSD2* | 22 | 24582041 | G | 1.015 | 0.9137 |

**Supplementary Table 3** Look-up of reported index SNPs shown to be associated to ALS through genome-wide association analysis. Common variants implicated through GWAS were looked up in single-variant testing of 1,196 cases and 608 controls performed with a logistic regression model correcting for ten principal components. Nominally significant (p < 0.05) associations are bolded. No association statistic for rs74654358:G>A was calculated in this analysis since this variant was observed as multi-allelic variant in our data (alleles: G, A, C). SNP refers to the dbSNP ID annotated to the reference and alternative allele on the “+” strand of the hg19 build of the human reference genome (for rs3849943:C>T this is hg19 chr9:g.27543382C>T). The closest gene to each SNP is annotated. CHR, chromosome; BP, base pair; A1, risk allele; OR, odds ratio.

| **Gene** | **T1**  **p-value** | **T5**  **p-value** | **MB**  **p-value** | **VT**  **p-value** | **SKAT**  **p-value** | **T1**  **MAC** | **T5**  **MAC** |
| --- | --- | --- | --- | --- | --- | --- | --- |
| *OPTN* | 0.796 | 0.141 | 0.595 | 0.313 | 0.111 | 8 | 93 |
| *HNRNPA1* | 0.723 | 0.723 | 0.725 | 0.862 | 0.515 | 4 | 4 |
| *PRPH* | 0.889 | 0.957 | 0.987 | 0.998 | 0.994 | 68 | 116 |
| *ATXN2* | 0.894 | 0.894 | 0.942 | 0.734 | 0.210 | 32 | 32 |
| *TBK1* | 0.586 | 0.181 | 0.631 | 0.484 | 0.192 | 28 | 152 |
| *ANG* | 0.994 | 0.994 | 0.963 | 0.948 | 0.590 | 6 | 6 |
| *SPG11* | 0.965 | 0.315 | 0.611 | 0.709 | 0.236 | 116 | 332 |
| *CCNF* | 0.683 | 0.563 | 0.523 | 0.921 | 0.801 | 14 | 92 |
| *FUS* | 0.545 | 0.545 | 0.545 | 0.670 | 0.545 | 5 | 5 |
| *PFN1* | 0.663 | 0.663 | 0.663 | 0.663 | 0.663 | 7 | 7 |
| *TARDBP* | 0.127 | 0.127 | 0.051 | 0.077 | 0.835 | 17 | 17 |
| *VAPB* | 0.325 | 0.325 | 0.282 | 0.599 | 0.601 | 25 | 25 |
| *SOD1* | 0.487 | 0.487 | 0.328 | 0.340 | 0.910 | 9 | 9 |
| *C21orf2* | 0.898 | 0.086 | 0.596 | 0.406 | 0.120 | 34 | 139 |
| *NEFH* | 0.191 | 0.191 | 0.106 | 0.014 | 0.306 | 57 | 57 |
| *CHCHD10* | 0.061 | 0.061 | 0.070 | 0.171 | 0.123 | 10 | 10 |
| *TUBA4A* | 0.086 | 0.086 | 0.086 | 0.086 | 0.226 | 2 | 2 |
| *DCTN1* | 0.558 | 0.537 | 0.319 | 0.398 | 0.490 | 64 | 110 |
| *ERBB4* | 0.075 | 0.075 | 0.120 | 0.144 | 0.031 | 18 | 18 |
| *ALS2* | 0.088 | 0.330 | 0.121 | 0.295 | 0.612 | 55 | 176 |
| *CHMP2B* | 0.067 | 0.067 | 0.067 | 0.067 | 0.760 | 6 | 6 |
| *NEK1* | 0.089 | 0.089 | 0.470 | 0.279 | 0.044 | 92 | 92 |
| *MATR3* | 0.332 | 0.332 | 0.405 | 0.587 | 0.509 | 17 | 17 |
| *SQSTM1* | 0.756 | 0.548 | 0.560 | 0.894 | 0.808 | 57 | 126 |
| *FIG4* | 0.541 | 0.724 | 0.442 | 0.586 | 0.899 | 25 | 120 |
| *GLE1* | 0.023 | 0.156 | 0.026 | 0.051 | 0.602 | 14 | 61 |
| *SETX* | 0.328 | 0.402 | 0.236 | 0.226 | 0.509 | 143 | 392 |
| *SIGMAR1* | 0.753 | 0.753 | 0.909 | 0.846 | 0.498 | 13 | 13 |
| *VCP* | 0.508 | 0.508 | 0.508 | 0.786 | 0.608 | 5 | 5 |
| *C9orf72* | 0.369 | 0.369 | 0.494 | 0.599 | 0.470 | 14 | 14 |

**Supplementary Table 4** Burden testing results for known ALS genes. A number of burden tests were performed on nonsynonymous and loss-of-function variants with < 5% frequency in 1 169 ALS cases and 608 controls. The following tests were performed: T1, test of variants with frequency <1%; T5, test of variants with frequency <5%; MB (Madsen-Browning) test inversely weights variants by frequency; VT (Variable Threshold), tests various frequency cut-offs and then performs multiple test correction; SKAT (Sequence Kernel Association Test), association test that allows for risk and protective variants. T1 and T5 MAC represent the total allele counts in the total sample at a minor allele frequency cut-off of 0.01 and 0.05 respectively. Results and full details of burden tests for all genes can be found at http://databrowser.projectmine.com/. If a known ALS gene does not appear here, it is because it does not contain nonsynonymous and/or loss-of-function variation in the gene at frequency <5%.

## Supplementary Methods

### Quality control

The full set of gVCFs were merged together by first converting the gVCFs to Plink format and then merging all files together. This generated a single dataset containing all variant sites across all individuals. Non-autosomal chromosome and multi-allelic variants were excluded from pilot analyses. Sample and SNP QC were performed using PLINK[^1,2^](https://paperpile.com/c/RpHGTX/6TRcb+9kJ6L) and VCFtools[^3^](https://paperpile.com/c/RpHGTX/FgJZ4). To begin sample QC, missingness by sample was calculated on a per-chromosome basis; all samples had missingness < 10% across all 22 chromosomes and no samples were removed at this step.

All other sample QC steps were performed on a set of high-quality biallelic SNPs that had minor allele frequency (MAF) > 10%, missingness < 0.1%, were LD-pruned at an r^2^ threshold of 0.2, were not A/T or C/G SNPs, did not lie in the major histocompatibility complex (MHC) or *LCT* locus, and did not occur in the inversions on chromosome 8 or chromosome 17. The ~30 000 SNPs overlapping this set of SNPs and HapMap 3 (HM3) were used to calculate principal components projecting the ALS cases and controls onto the HM3 samples. Samples of non-European ancestry, defined as further than 10 standard deviations from the European-ancestry populations in HM3 (CEU, people of Northern and Western European ancestry living in Utah; TSI, Tuscans in Italy), were excluded from analysis to ensure an ancestrally homogeneous group of samples for association testing. Samples with an inbreeding coefficient > 3 s.d. from the mean of the distribution were excluded, as were unexpected related samples. Genotypes available from genotyping on the Omni 2.5M array were compared to sequencing genotypes, and samples with < 95% concordance were dropped from the analysis. Lastly, samples with discordant sex information (comparing chromosome X genotypes and phenotype information) were excluded.

For variant QC, variants with missingness > 5% were removed, as were variants out of Hardy-Weinberg equilibrium in controls (p < 1 × 10^-6^) and monomorphic variants (induced by sample exclusions). Differential missingness between cases and controls was checked and variants with p < 1 × 10^-6^ were removed. Variants with extreme depth of coverage (> 6 s.d. from the mean of the total depth distribution) were also excluded. Finally, the mitochondrial, X and Y chromosomes were excluded from analysis (but will be included in later analyses as sample sizes in Project MinE continue to grow). Approximately 10 million sites were lost during variant QC.

For simulation analyses, samples were filtered down to all those samples with complete birthplace information born in The Netherlands (N = 1 132 cases and N = 569 controls).

### Geographical mapping

For cases and controls, birthplaces were mapped using latitude and longitude GPS coordinates obtained from an online geocode converter (www.gpsvisualizer.com) and a Google Maps API. For all non-Dutch birthplaces, we manually checked the conversion from free text to GPS coordinates to correct translation of spelling mistakes. Geographical distances between individuals in kilometres were calculated using the Haversine formula. To compare population genetic effects with those previously reported, we grouped individuals according to the provinces they were born in.

### Principal component analysis

To calculate principal components (PCs), a pruned set of high quality SNPs with a genotyping rate 0.98 were included and SNPs within the MHC or *LCT* locus or in the inversions on chromosome 8 or chromosome 17 were excluded. To assess the contribution of rare variants, different minor allele frequency (MAF) thresholds were used for different rounds of PC calculations. A genetic relationship matrix and the subsequent eigenvectors of the first 100 principal components were calculated using genome-wide complex trait analysis (GCTA)[^4^](https://paperpile.com/c/RpHGTX/ymAiA).

### Identity-by-descent analysis.

All non-singleton variants were phased using SHAPEIT2[^5^](https://paperpile.com/c/RpHGTX/ex1YX). Subsequently BEAGLE4[^23^](https://paperpile.com/c/RpHGTX/MDo7q) was used to detect runs of identity by descent (IBD) between individuals. The hg19 recombination map obtained from the 1000 Genomes Projects was used to transform genetic positions from basepairs to centimorgans (cM). IBD segments shorter than one cM were excluded and regions with excessive IBD were excluded after visual inspection.

### Association testing in ALS cases and controls

We tested both common and rare variants for an association with ALS risk. Genotypes at common variants (MAF > 0.5%) were tested for an association with case/control status using logistic regression (PLINK v1.9)[^1,2^](https://paperpile.com/c/RpHGTX/6TRcb+9kJ6L) assuming an additive model. Optionally, the first 10 principal components were included as covariates.

To test rare variation, we performed burden testing. All variants were functionally annotated using ANNOVAR[^7^](https://paperpile.com/c/RpHGTX/Q2A43). We then determined three functional groups for gene-based association testing: (a) loss of function (LOF) variants (premature stop variants, stop-loss variants, variants at splice sites, and frameshift indels), (b) nonsynonymous variants, and (c) LOF and nonsynonymous variants (aggregated). Burden testing was implemented using ScoreSeq[^8^](https://paperpile.com/c/RpHGTX/SFadK) and was performed across all variants with MAF < 1%. All burden tests were adjusted for sex and PCs 1 – 10. We performed the following burden tests: T1, Variable Threshold, Madsen-Browning (variants are inversely weighted by frequency), and SKAT. The first three burden tests test for variants of all the same direction of effect in a single locus; the SKAT test allows for variants in a single locus to have multiple directions of effect.

To assess the effect of geographically matched cases and controls, we simulated 100 phenotypes under the null-hypothesis of no genetic association for the traits in two different scenarios: (1) perfect matching of cases and controls, which is equal to 100 permutations and (2) imperfect matching in which we varied the number of cases and controls drawn from specific geographic regions following a North-to-South gradient (**Supplementary Figures 1,6**). We performed genome-wide burden testing (T1, MB, VT and SKAT) for each phenotype (2 × 100 simulated phenotypes) and for both covariate scenarios. The most extreme p-value from each simulation was extracted (resulting in 100 extreme p-values total, per scenario); the fifth-most extreme p-value represented the p-value threshold necessary to maintain type I error at 0.05 in burden testing.

### Association testing in ALS cases and externally sequenced controls

The ALS cases were merged together from unrelated (N = 498) individuals who were whole-genome sequenced as part of the Genome of the Netherlands (GoNL) project. GoNL contains approximately 250 trios selected that were randomly ascertained from the Dutch population. Samples were sequenced at ~14x coverage on the Illumina HiSeq 2000. Variant sites observed in GoNL only were ignored. Variant sites observed in ALS but not in GoNL were set as monomorphic for the reference allele across all GoNL samples.

We calculated principal components in the merged set of ALS cases and GoNL controls, using an LD-pruned (r^2^ = 0.2) set of SNPs with MAF > 10% and missingness < 0.1%, and excluding the following: variants in the MHC or the *LCT* locus, A/T and C/G variants, variants falling in inversions on chromosomes 8 and 17, and any variants with a frequency difference > 5% when comparing the ALS and GoNL samples (likely sequencing errors). We then performed single-variant testing, comparing the ALS cases with GoNL controls. Two sets of single-variant testing were performed, one with inclusion of the top ten PCs and one without. Similarly, we performed burden testing comparing the ALS cases and GoNL controls, optionally including the top 10 PCs as covariates.

## References

1 [Purcell S, Neale B, Todd-Brown K *et al.* PLINK: a tool set for whole-genome association and population-based linkage analyses. *Am J Hum Genet* 2007; **81**: 559–575.](http://paperpile.com/b/OCy5SL/QsAJ4)

2 [Chang CC, Chow CC, Tellier LC, Vattikuti S, Purcell SM, Lee JJ. Second-generation PLINK: rising to the challenge of larger and richer datasets. *Gigascience* 2015; **4**: 7.](http://paperpile.com/b/OCy5SL/L1mLC)

3 [Danecek P, Auton A, Abecasis G *et al.* The variant call format and VCFtools. *Bioinformatics* 2011; **27**: 2156–2158.](http://paperpile.com/b/OCy5SL/JKLdm)

4 [Yang J, Lee SH, Goddard ME, Visscher PM. GCTA: a tool for genome-wide complex trait analysis. *Am J Hum Genet* 2011; **88**: 76–82.](http://paperpile.com/b/OCy5SL/RqJpj)

5 [Delaneau O, Marchini J, Zagury J-F. A linear complexity phasing method for thousands of genomes. *Nat Methods* 2012; **9**: 179–181.](http://paperpile.com/b/OCy5SL/Gcbq4)

6 [Browning BL, Browning SR. Improving the accuracy and efficiency of identity-by-descent detection in population data. *Genetics* 2013; **194**: 459–471.](http://paperpile.com/b/OCy5SL/roRkg)

7 [Wang K, Li M, Hakonarson H. ANNOVAR: functional annotation of genetic variants from high-throughput sequencing data. *Nucleic Acids Res* 2010; **38**: e164.](http://paperpile.com/b/OCy5SL/fOx3r)

8 [Lin D-Y, Tang Z-Z. A general framework for detecting disease associations with rare variants in sequencing studies. *Am J Hum Genet* 2011; **89**: 354–367.](http://paperpile.com/b/OCy5SL/5CAUe)
